# Biodegradable gelatin-based films with nisin and EDTA that inhibit *Escherichia coli*

**DOI:** 10.1371/journal.pone.0264851

**Published:** 2022-03-10

**Authors:** Romina L. Abarca, Javiera Medina, Nancy Alvarado, Pablo A. Ortiz, Bernardo Carrillo López

**Affiliations:** 1 Departamento de Ciencias Animales, Facultad de Agronomía e Ingeniería Forestal, Pontificia Universidad Católica de Chile, Macul, Santiago, Chile; 2 Instituto de Ciencia y Tecnología de los Alimentos, Facultad de Ciencias Agrarias, Universidad Austral, Valdivia, Chile; 3 Instituto de Ciencias Químicas Aplicadas, Facultad de Ingeniería, Universidad Autónoma de Chile, San Miguel, Santiago, Chile; 4 Núcleo de Química y Bioquímica, Facultad de Estudios Interdisciplinarios, Universidad Mayor, Santiago, Chile; Universidade Estadual de Ponta Grossa, BRAZIL

## Abstract

In this study, we developed gelatin-based films for active packaging with the ability to inhibit *E*. *coli*. We created these novel biodegradable gelatin-based films with a nisin-EDTA mix. FT-IR, TGA, and SEM analysis showed that nisin interacted with the gelatin by modifying its thermal stability and morphology. The use of nisin (2,500 IU/mL) with concentrations of Na-EDTA (1.052 M stock solution) distributed in the polymer matrix generated a significant decrease in the growth of *E*. *coli* when compared to the control. In freshly made films (t_0_), the growth of *E*. *coli* ATCC 25922 was reduced by approximately 3 logarithmic cycles. Two weeks after the films were made, a reduction in antimicrobial activity was observed in approximately 1, 1 and 3 logarithmic cycles of the films with 5%, 10% and 20% of the compound (nisin/Na-EDTA) distributed in the polymer matrix, respectively. This evidences an antimicrobial effect over time. Also, biodegradation tests showed that the films were completely degraded after 10 days. With all these results, an active and biodegradable packaging was successfully obtained to be potentially applied in perishable foods. These biodegradable, gelatin-based films are a versatile active packaging option. Further research on the barrier properties of these films is needed.

## Introduction

One of the major challenges for the food industry, particularly for the perishable food processing industries—such as the meat and dairy industries—is conservation, that is, avoiding the proliferation of microorganisms that break down food, generating economic losses and serious damage to consumers’ health. Currently, competition in the food industry is very high and any company that does not offer quality in its products is doomed to failure. The current consumer is increasingly demanding, thus, the industry is making daily efforts to guarantee the quality and safety of the manufactured products [1, 2].

For example, meat—as food and source of proteins of high biological value—has required many techniques for its processing, storage, and preservation since man discovered fire and learned to cook it for consumption. Given its high perishability condition caused by its high water content, it requires different processes for its conservation that, over time and depending on technological availability, have been changing and improving its shelf life [3, 4]. Microorganisms that alter meat reach it by infection of the living animal (endogenous contamination) or postmortem invasion (exogenous contamination); although both are of great importance, alteration of meat as a result of exogenous contamination is the most frequent, thus, the consumer can suffer serious infections or intoxication from consuming meat from contaminated animals [5]. *Escherichia coli*, the causative agent of toxinfection, can produce a toxin that invades the host’s intestine. Some products are handled under poor hygiene standards; the presence of this microorganism in meat and meat products indicates a deficiency in the quality assurance program [6]. This last microorganism is one of the most recurrent concerning contaminations within the meat industry [7–9]. One of the ways to preserve the shelf life of perishable foods is through packaging systems. A packaging is defined as a structure intended to contain a product that protects it from microbiological damage and changes in organoleptic characteristics, acts as a marketing element, and is a passive barrier since it only serves to isolate food from the environment [10–12]. The need for improving preservation and extend the shelf life of food has led to the need for innovating through a new packaging concept: active packaging. This packaging has components incorporated in its polymer matrix that can release or absorb substances from or to the food, allowing it to extend its shelf life and preserve the characteristics of the food. The active components of the container can generate changes at the organoleptic or chemical level, so it’s fundamental to take care that it remains within the regulations of each country [13].

The most common materials used for food packaging are paper, glass, aluminum, and plastic based on polymers from non-renewable (petroleum-based) sources [14]. These materials have excellent physical and mechanical properties, with a wide range of applications due to their low cost and easy processing [15]. Although these materials offer advantages, the waste produced by their processing over time has accumulated in massive proportions, generating a negative impact on the environment. Therefore, these containers have become a great threat to the environment due to their low biodegradation capability and high waste flow [3, 16]. Given this problem, various strategies have been proposed to reduce the environmental impact generated by conventional packaging, such as the possibility of using biodegradable polymers, which have become a focus of interest in recent years due to the exhaustion of fossil fuels and then need for the development of food packaging with specific desired properties and with less impact on the environment [17, 18]. Among these biodegradable materials, carbohydrates, lipids, proteins, among others, have been used to develop films with increasingly versatile properties. Protein-based films offer better mechanical and barrier properties due to both the structural specificity of the proteins and the ability to form stronger intermolecular covalent bonds [19]. Biopolymers—such as gelatin—have emerged as an effective alternative to conventional packaging materials due to their biocompatibility [20], good film-forming properties, abundance in nature, effective absorption of UV light due to the presence of aromatic amino acids in their structures, and good mechanical properties; however, they are sensitive to humidity and have poor barrier properties against water steam [21, 22].

In this regard, it would be interesting to link both technologies and obtain food packaging with antimicrobial capacity and a high percentage of biodegradability in short times, generating less environmental impact [23]. The choice of which active agent to use becomes relevant; it will be a direct part of the polymeric matrix of the packaging which must extend the shelf life of the food, guaranteeing the safety of packaged products [24], and not generate waste in the degradation process of the base material. Among the agents with antimicrobial capacity permitted by current legislation, silver, silver zeolite, glucose oxidase, chlorine dioxide, natamycin, sulfur dioxide, allyl isocyanate, and nisin stand out [1, 25, 26]. The latter has great potential and effectiveness when used in the food industry and is defined as an antimicrobial protein or peptide of ribosomal synthesis produced by lactic acid bacteria, which are used at low concentrations to improve the quality and preservation of food. Besides, it presents effective microbiological inhibition against pathogenic microorganisms involved in foodborne illness [27]. Nisin is produced by the microorganism *Lactococcus lactis* subsp. Lactis, classified as lantibiotics (class I), has a wide spectrum of inhibition. A peptide made up of 34 amino acids is classified as Generally Recognized Safe (GRAS) by the FDA and its use as a food preservative (E234) has been allowed by the European Union since 1983, however, it does not act against Gram-negative bacteria, yeasts, or fungi. This substance, in combination with chelating agents (organic acids, EDTA) or emulsifying agents (Tween 80), shows a great inhibitory activity against Gram-negative bacteria since the protective cell membrane is altered allowing the formation of pores and, as a result of that, the ion output and loss of the proton matrix force are caused, thus generating cell death [28, 29]. In combination with nisin, for example, prior studies have shown this nisin-EDTA combination to be effective against other gram-negative bacteria such as *Salmonella typhimurium* [30].

The use of nisin in antimicrobial active films is not new, given that nisin is one of the most commonly used bacteriocins in active packaging [31]. Although the chelator EDTA has been combined with bacteriocins to potentiate their effects in active antimicrobial packaging, specifically, a gelatin-based biofilm with nisin-EDTA has not been reported in the recent literature. Based on the background presented in this study, highly biodegradable edible gelatin films were made with a mixture of nisin/Na-EDTA, obtaining biodegradable composites with antibacterial capacity against *Escherichia coli*.

## Materials and methods

### Materials

Edible gelatin (220 bloom) was used as the polymer matrix to produce the films, which was purchased from the Floramatic company, Santiago, Chile. The antimicrobial agent nisin (C_143_H_230_N_42_O_37_S_7_, CAS1414-45-5) was purchased from Chr. Hansen Holding A/S, Denmark. *Escherichia coli* ATCC 25922 strain. Culture medium Agar soy trypticase was purchased from Becton, Dickinson, and Company and EC broth was produced by the Liofilchem SRL company, Italy. Glycerol (C_3_H_8_O_3_) was purchased from the Winkler company, Santiago, Chile. Ethylenediaminetetraacetic acid, tetrasodium salt (Na-EDTA, No. CAS64-02-8) was purchased from the GTM company, Argentina.

### Preparation of nisin in solution

100 mg of nisin were diluted in 10 mL of 0.02 M HCL (stock solution with a concentration of 10 mg/mL) of initial 10,000 IU/mL concentration at pH 2. The solution was filtered using a 0.22 μm filter and left in 1 mL aliquots frozen at -18°C as stock. Different concentrations were prepared from the nisin stock solution in combination with 40% Na-EDTA, (1500, 2000 and 2500 IU/mL).

### Minimum Inhibitory Concentration (MIC)

The determination of the MIC of nisin against the *E*. *coli* ATCC 25922 strain was carried out using the microdroplet technique in sterile Petri dishes. The plates were filled with 12 mL of soy trypticase agar, allowed to solidify at room temperature. Then, 100 uL of the known bacterial solution (1x10^6^ CFU/mL) seeded by extension were applied with a sterile glass rake. Subsequently, 20uL microdroplets of the nisin/EDTA solution were applied in each of the concentrations indicated in point 2.2 in triplicate. The plates were incubated for 24 hours at 37°C. Results were reported in accordance with the formation of inhibition halos around the microdroplet (diameter).

### Film preparation

The casting technique was used to make the antimicrobial films. Four different gelatin-based solutions were prepared with different percentages (0, 5, 10, and 20%) of the nisin/Na-EDTA solution, with a concentration of 2500 IU/mL, in accordance with the value obtained from MIC.

For each formulation of the films produced, edible gelatin powder (220 bloom) was dissolved in 10% distilled water, as well as glycerol plasticizer by the same percentage; the amount of water and the solution with the active agent (nisin) varied among each formulation. They were shaken on a hot plate (Scilogex model MS-H280-Pro) at 60°C for 120 minutes, with constant stirring at 1000 rpm. Finally, each solution was poured into 22 cm-diameter glass Petri dishes and brought to the oven for solvent evaporation (distilled water) for 48 hours at 45°C. After this time, they were stored in desiccators with humidity control, thus obtaining a control film (**CF**): 10% edible gelatin, 10% glycerol, 80% distilled water; active film 1 (**AF1**): 10% edible gelatin, 10% glycerol; 75% distilled water and 5% nisin/Na-EDTA solution; active film 2 (**AF2**): 10% edible gelatin, 10% glycerol; 70% distilled water and 10% nisin/Na-EDTA solution; active film 3 (**AF3**): 10% edible gelatin, 10% glycerol; 60% distilled water and 20% nisin/Na-EDTA solution.

### Film characterization

Each of the films is characterized in order to know which presents the best antibacterial activity against the target microorganism.

#### Thermal assay

**Thermogravimetric analysis (TGA)**. TGA was performed in accordance with the methodology described by Abarca et al. [32], with modifications. It was carried out using TGA/DSC1 STARe System Metler Toledo (Greifensee, Switzerland) thermo-gravimetric analyzer. Approximately 20 mg of sample were uniformly spread on the bottom side of alumina crucible. The experiment was performed at a heating rate of 10°C/min in a dynamic high purity nitrogen flow of 50ml/min. The furnace temperature was programmed to rise from 10 to 900°C.

#### Optical properties

The optical properties assessment was performed in accordance with the methodology described by Abarca et al. [32]. Film colour was measured using a colorimeter Hunterlab MiniScan XE Plus 45/0-L. The CIELAB scale was used, and the lightness (L*) and chromaticity parameters a* (red–green) and b* (yellow–blue) were measured. A standard white colour plate (L* = 97.11, a* = −0.03, and b* = 1.96) was used as the background for colour measurements, and D65 illuminant and 2° observer were used for analyses. Each analysis was replicated 20 times, and the results reported the average value. Colour differences (ΔE) were calculated using the Equation [32]:

$$\Delta \text{E}=[\left(\Delta {\text{L}}^{*}\right)2+\left(\Delta {\text{a}}^{*}\right)2+\left(\Delta {\text{b}}^{*}\right)2]1/2$$ (1)

Opacity of the films was determined using a UV–visible spectrophotometer (Spectronic® Genesys 5). The film samples were cut into rectangular pieces (1.0 × 4.5 cm) and placed on the sample compartment of a spectrophotometer. An empty compartment was used as a blank reference for the measurement. Opacity of the films was calculated using the Eq (2)

$$\text{Opacity}=\text{Abs}\;600/\text{X},\;\text{where}\;\text{Abs}600=\text{absorbance}\;\text{at}\;600\;\text{nm}\;\text{and}\;\text{X}=\text{film}\;\text{thickness}\;\left(\text{mm}\right).$$ (2)

#### Fourier transform infrared (FT-IR) spectroscopy

FT-IR was used to identify the chemical structure of the composite films and possible interactions between their components. The FTIR spectra of the membranes were measured with a Jasco FT/IR6200 spectrophotometer. The spectra were determined as the average of 64 scans recorded at a resolution of 4 cm^-1^ in the range from 4000 to 400 cm^-1^.

#### Thickness and morphology

With the aim to determine the influence of the nisin on the thickness of films, the thicknesses of the different films were measured using a Mitutoyo Digimatic micrometer (ID-C112 model, Kawasaki, Japan). The measurements were carried out in fifteen different points of each obtained film. Thus, an average thickness value with its corresponding standard deviation for different films is reported. In order to evaluate the effect of the presence of nisin on the morphology of the films, SEM micrographs of the films were obtained by scanning electron microscope CrossBeam (FIB-SEM), AURIGA Compact con software SmartSEM.

#### Biodegradability of the films

The film degradation test was performed in accordance with the methodology described by de Oliveira et al. [18], with modifications. Natural organic soil contained in plastic boxes was used as the degradation environment. The film samples were cut into rectangles (2cm×3cm), dried at 60°C to a constant weight (w_0_), placed in a plastic mesh, and buried 5 cm below the soil surface. The soil was watered to maintain a moisture level of approximately 65%. The film was analyzed after 10 days (w_10_) and the rate degradation was calculated by the Equation.


$$Rate\;Degradation=\left(\frac{\omega_{0}-\omega_{10}}{\omega_{0}}\right)x100.$$ (3)

#### Antimicrobial activity of the films

The antibacterial evaluation against Escherichia coli was performed based on ASTM E2149-10 with minor modifications. Standard Test Method for Determining the Antimicrobial Activity of Immobilized Antimicrobial Agents Under Dynamic Contact Conditions. The test involves determination of a variable number of bacteria in liquid medio with samples (film) and comparison with the number of bacteria in a solution without samples.

A preinoculum was prepared by transferring bacterial colonies with a handle loop in a test tube with 5 mL of Escherichia coli (EC) broth. The test tube was incubated for 16 h at 3°C and constant agitation. After this time, 1 mL of the bacterial suspension was taken and transferred to a test tube with 15 mL of EC medium to obtain inoculum. It was incubated at 37°C in a shaking incubator, until the culture was found in 0.8 optical density at 600 nm. In this range the bacterial cells are in exponential phase. Then, diluting fixed concentration prepares concentration of 5×10^6^ UFC mL^−1^ of the microorganism in a phosphate buffer stock solution of KH2PO4 0.3 mM with pH = 7.2±0.1. A 0.5 g sample was placed in 250 ml Erlenmeyer flasks and covered with 50±0.1ml of the bacteria-containing buffer. Flasks were placed in a shaker thermostated at 37°C and subjected to shaking for 24 h. A bacteria suspension without samples was used as reference. 1 ml of suspension was collected after the incubation, and the number of bacteria was determined using the decimal dilution method, followed by pour plate method on the TSA, in triplicate from each of the dilutions. After a 24-hour at 37°C colonies were counted, and the number of bacteria was calculated expressed as log_10_ bacteria reduction.

### Statistical analysis

The experiment was replicated three times. In each replication, analyses were conducted in triplicate. The results correspond to the mean ± standard deviation of the mean. Data were analyzed by one-way analysis of variance (ANOVA), whereas Fisher’s test (p< 0.05) was used for testing differences between the means.

## Results

### Nisin/EDTA MIC against *Escherichia coli*

Normally, nisin has activity against Gram positive bacteria, but low activity against Gram negative bacteria. In the second case, it has been described that when combining nisin with chelating agents such as Na-EDTA in concentrations higher than 100μM, an evident increase in the inhibition effects of gram-negative bacteria is observed [33]. The nisin/Na-EDTA mixture showed antibacterial effect in 6 of the 9 concentrations studied (Table 1). The table presents three divisions because three different concentrations of Na-EDTA were used (3 treatments), of which only two had the desired effect.

**Table 1:** Determination of the minimum inhibitory concentration of the nisin/Na-EDTA mixture against *E*. *coli*.*

Active agent mixture	Inhibition halo (mm)
1500 UI/mL/Na-EDTA 0.0126 M	0^a^
2000 UI/mL/Na-EDTA 0.0126 M	0^a^
2500 UI/mL/Na-EDTA 0.0126 M	0^a^
1500 UI/mL/Na-EDTA 0.0253 M	6.3 ± 0.57^a^
2000 UI/mL/Na-EDTA 0.0253 M	8.3 ± 0.5^b^
2500 UI/mL/Na-EDTA 0.0253 M	10.0 ± 0.5^c^
1500 UI/mL/Na-EDTA 1.052 M	16.5 ± 1.2^a^
2000 UI/mL/Na-EDTA 1.052 M	21.0 ± 2.1^b^
2500 UI/mL/Na-EDTA 1.052 M	28.0 ± 1.0^c^

* Each value represents the mean of 3 replicates with the corresponding standard deviation. Data were statistically analysed with multiple range test, using the method of least significant difference (LSD) Fisher. Different lowercase letters denote statistically significant differences (p < 0.05).

The results show that the first three analyzed mixtures of nisin (1500, 2000, 2500UI/mL and Na-EDTA (0.0126M), showed no inhibition halos. The following analyzed mixtures (Nisin + Na-EDTA (0.0253M)) against *E*. *coli* presented inhibition halos, which were compared with the effectiveness of three proven antibiotics against *E*. *coli* as a model to determine levels of resistance and sensitivity of the microorganism against the active mixture of nisin/Na-EDTA. In this case, they were compared with the ampicillin (20 mcg; halo ≤13mm resistant; halo ≥17 susceptible), ceftazidime (30 mcg; halo ≤17mm resistant; halo ≥21mm susceptible), and tetracycline (25 mcg; halo ≤11mm resistant; ≥15mm susceptible) antibiotics [34, 35]. Despite presenting inhibition halos, these are not sufficient to generate susceptibility in the target microorganism. Then, by maintaining the nisin concentration and increasing the molarity of Na-EDTA, higher inhibition halos are obtained, thus presenting a minimum inhibitory concentration of 2500UI/mL of nisin and 1.052M Na-EDTA, whose halo formed was of 28 ± 1.0 mm. This concentration and mixture were chosen as MIC because the diameter of the inhibition halo is above the halos generated by antibiotics against *E*. *coli*. On the other hand, Sangcharoen et al. [36] reported similar results against *Salmonella enteritidis* ATCC 13076 when combining the same concentrations based on the active agent (nisin); in this regard, it is relevant to consider that *Salmonella enteritidis* is also a Gram-negative microorganism, which shows the effectiveness of Na-EDTA as an agent that enhances the activity of nisin.

### Characterization of the films

#### Films TGA

**Fig 1:**
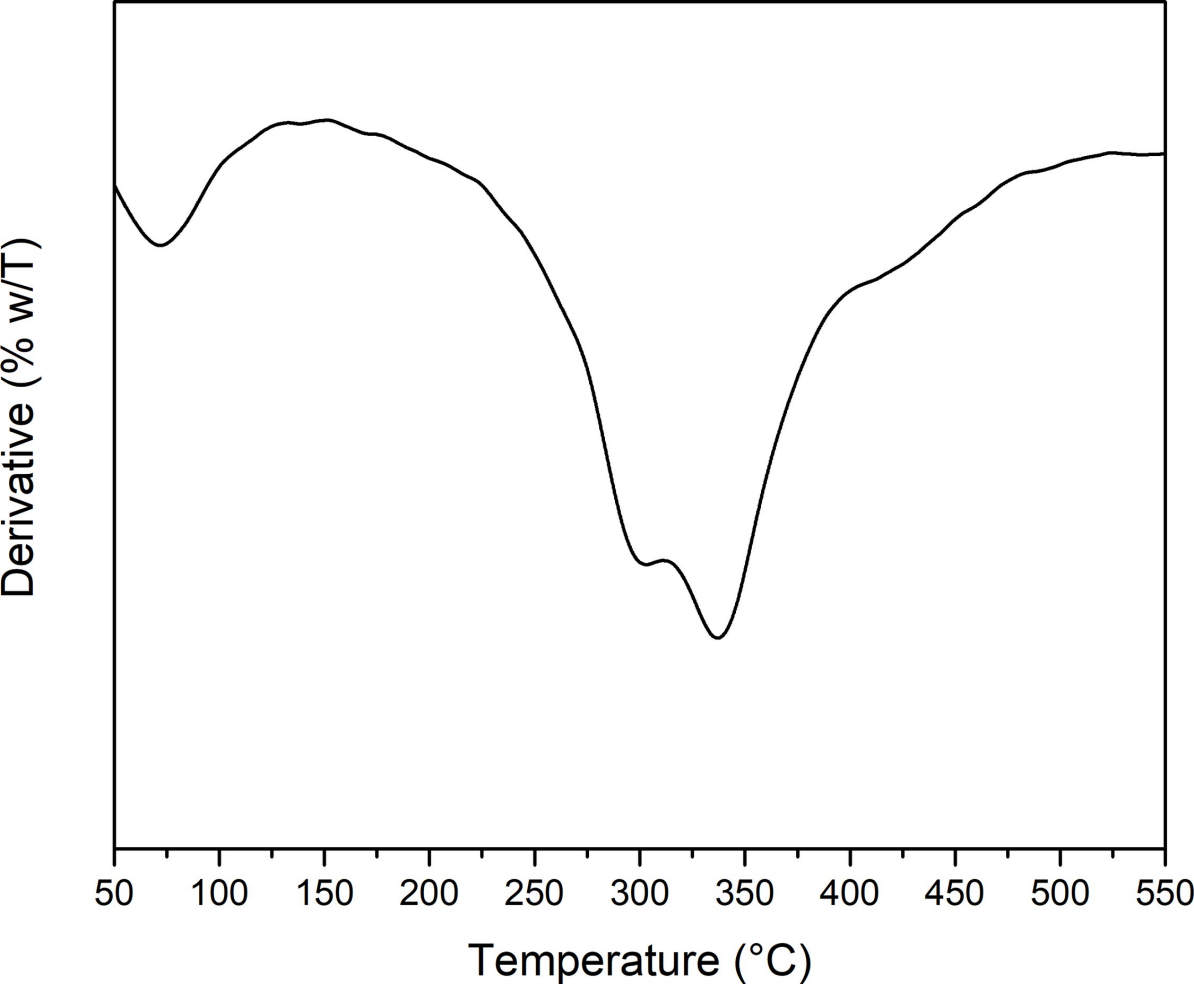
DTG thermogram of nisin.

**Fig 2:**
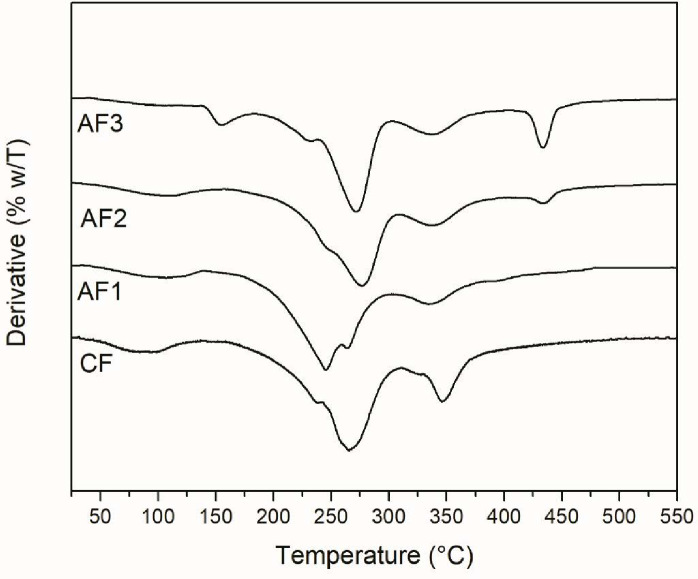
DTG thermograms of gelatin films studied. AF: Active Film, CF: Control Film.

TGA analyses of different gelatin-based films were carried out in order to reveal the thermal degradation behavior. Figs 1 and 2 shows the DTG thermograms for nisin and all the gelatin-based films obtained, respectively In general, four main stages of weight loss are observed in Fig 2. The first one, around 100°C, corresponds to the evaporation of free or adsorbed water of the films. The second stage, around 230–240°C, is observed as a shoulder from the major peak, which is attributed to the loss of glycerol that was used as plasticizer [37, 38]. The major thermal decomposition occurred in the third stage, around 246–276°C, which corresponds to the gelatin matrix [39]. Regarding CF film, the major decomposition was at 265°C, while for AF2 and AF3 films, this temperature increased to 276°C and 272°C, respectively. As for AF1 film, a slight decrease in the major decomposition at 246°C is observed. For AF2 and AF3 samples at 420°C a decomposition can be observed. This could be explained due to the content of nisin incorporated in those films since Fig 1, shows the decomposition of nisin where is possible to observe at 420°C a small shoulder. These results suggest that the nisin/Na-EDTA addition at the indicated concentrations (AF2 and AF3), increased the thermal stability of the gelatin films. This could be explained through the intermolecular interactions developed as hydrogen bonding as both gelatin and nisin have functional groups capable of generating these interactions. In the case of AF1 film, the concentration of nisin/Na-EDTA incorporated does not seem to be enough for increasing the thermal stability in gelatin film. The fourth stage, around 335–340°C, can be attributed to high molecular weight protein fraction.

#### Optical properties

The optical properties are relevant when choosing a food material since consumer appreciation of the product plays a primary role in their choice. The colour of the different films made was determined using the CIELab scale, where L* corresponds to luminosity (0-black and 100-white) and a* and b* correspond to color parameters whose distribution is green(-)/red(+) and blue(-)/yellow(+), respectively [18].

The L* values did not show variations after the addition of the antimicrobial agent with respect to the CF (p> 0.05) (Table 2). This suggests that the films tend to white despite the increase in the concentration of the agent and that they do not differ between films, therefore there are no statistically significant differences (p> 0.05). This agrees with the findings of Kumar et al. [40], who reported that when adding silver nanoparticles to a film composed of chitosan, gelatin, and polyethylene glycol, they do not differ and tend to be white.

**Table 2:** Analysis of color and opacity of elaborated films.**

Samples	a*	b*	L*	ΔE	Opacity
**CF** †	-2.73 ± 0.07^a^	5.53 ± 0.2^a^	87.18 ± 0.83^a^	-	0.15 ± 0.03^a^
**AF1** ‡	-2.78 ± 0.15^a^	5.47 ± 0.1^a^	87.75 ± 0.65^a^	0.71 ± 0.49^a^	1.14 ± 0.12^b^
**AF2** §	-3.04 ± 0.11^b^	6.55 ± 0.14^b^	87.18 ± 2.27^a^	1.96 ± 1.73^a^	0.92 ± 0.13^b^
**AF3** ¶	-3.1 ± 0.1^b^	6.44 ± 0.09^b^	87.65 ±0.69^a^	1.24 ± 0.25^a^	1.06 ± 0.19^b^

** Each value represents the mean of 10 replicates with corresponding standard deviation. Data were statistically analysed with multiple range test, using the method of least significant difference (LSD) Fisher. Different lowercase letters denote statistically significant differences (p < 0.05).

† CF: Control film (10% edible gelatin, 10% glycerol, and 80% distilled water).

‡ AF1: Active film 1 (10% edible gelatin, 10% glycerol; 75% distilled water and 5% nisin/Na-EDTA solution).

§ AF2: Active film 2 (10% edible gelatin, 10% glycerol; 70% distilled water and 10% nisin/Na-EDTA solution).

¶ AF3: Active film 3 (10% edible gelatin, 10% glycerol; 60% distilled water and 20% nisin/Na-EDTA solution).

The results show that the addition of the active agent in the chromatic parameter a* tended towards the green color, showing significant differences between CF, AF2, and AF3. Regarding the color parameter b*, it was observed that all the films tend to yellow, being observed, as in the previous case, statistically significant differences of AF2 and AF3 with respect to CF. This is due to the increase in nisin in the polymeric matrix, considering that CF does not have nisin, and AF2 and AF3 have increasing nisin respectively. The colour difference (ΔE) of the films did not show statistically significant variations between them or concerning the control sample (p> 0.05).

When choosing a food packaging material, the opacity parameter is relevant since, in most cases, a higher opacity is undesirable as it hinders the correct visualization of the state of the food by the potential consumer [41]. In some applications, it is desirable to provide protection against reactions from the deterioration produced by the effect of light [42].

When visually appreciating the elaborated films, they are transparent. When performing the opacity analysis on each of the analyzed samples, it was possible to observe that the opacity parameter increased with the incorporation of the active solution when compared to CF, denoting statistically significant differences (p <0.05), while the analysis conducted between the elaborated active films showed no differences regarding the studied parameter. Cazón et al. [14] point out that the differences in opacity or transparency of the films are mediated by the thickness of each one of them, which is corroborated by the values obtained here.

#### FT-IR

FT-IR is a powerful tool that was used in this study to confirm the presence of gelatin and nisin and which, through displacement in bands, can deliver information about possible interactions within the films. Since the chemical structure of both gelatin and nisin is constituted by amino acid residues, its FT-IR signals are similar.

**Fig 3:**
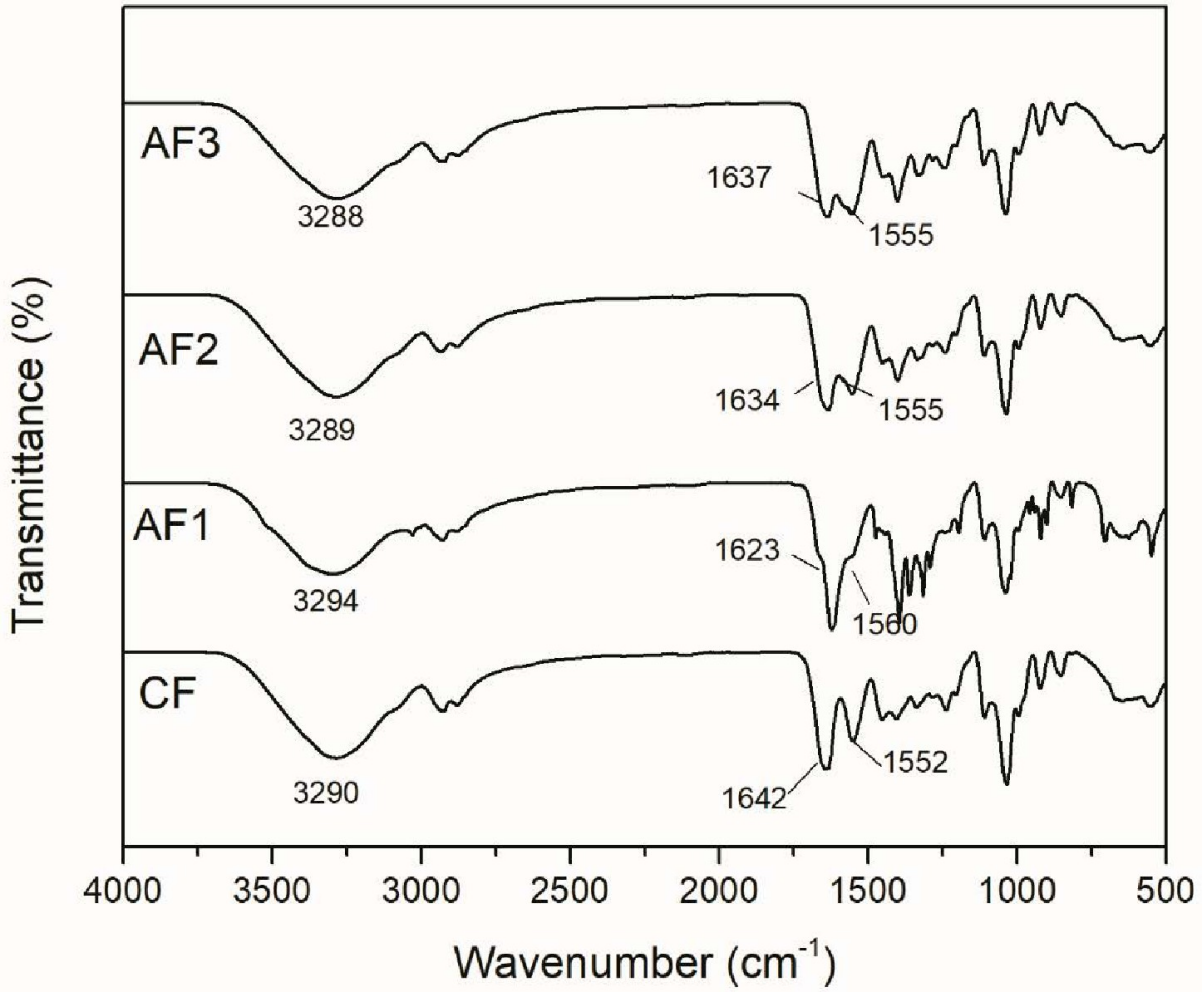
FT-IR of gelatin films studied. AF: Active Film, CF: Control Film.

The FT-IR spectrum of gelatin (Fig 3) shows that its characteristic peaks correspond to those of a complex protein, a polymer constituted by amino acid units. The gelatin spectrum has been analyzed in other studies [39, 43, 44]. The main signals in the spectra of CF show a band at 3290 cm^-1^ named amide A, corresponding to the N-H stretching overlapping O-H stretching vibration. The amide I band is identified at 1642 cm^-1^, which represents C = O stretching of proteins amide groups. The peak at 1552 cm^-1^, named amide II, is assigned to N-H stretching of protein ([37, 38, 45]).

**Fig 4:**
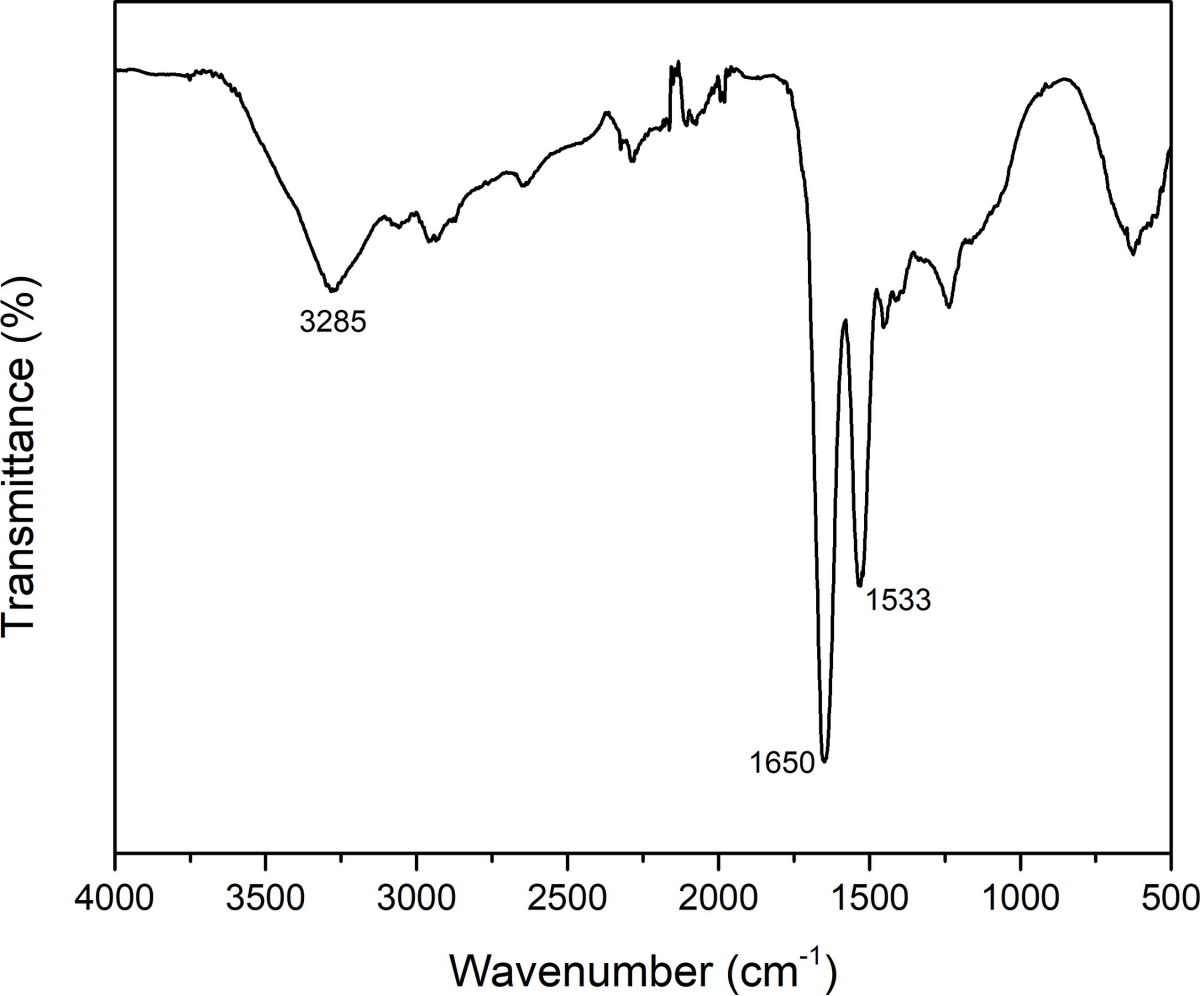
FT-IR of nisin.

Nisin spectrum (Fig 4) revealed a band at 3285 cm^-1^ corresponding to OH stretching (in COOH group), a peak assigned to amide I at 1650 cm^-1^, while at 1533 cm^-1^ a band identified as amide II was observed [46–48]. The FT-IR spectra of AF1, AF2, and AF3 films show a shift in the characteristic bands of functional groups capable of forming intermolecular interactions, such as hydrogen bonding. Displacements are observed in amide A, amide I, and amide II bands of both CF and nisin. The FT-IR supports the evidence that nisin was incorporated successfully into the gelatin films and also showed that there is compatibility between them.

#### Determination of thickness and morphology of the films

*Thickness of the films*. The thickness of the films ranged from 0.57 to 0.75 mm. The incorporation of the active agent increased the thickness of films AF1, AF2, and AF3, by 0.06, 0.14, and 0.18 mm, respectively, when compared to CF (Table 3). This increase generated significant differences (p <0.05) in the films with 10% (AF2) and 20% (AF3) of the active agent, probably due to an increase in the solids content after the incorporation of the antimicrobial agent into the formulation [18]. The recently described event—regarding the variations and increases of the thickness of the films—directly affects the opacity parameter, as the greater the thickness, the greater the opacity value obtained; therefore, a film with a low level of transparency could be intended for use as a packaging system for photosensitive food [49, 50]. The biodegradability process of films is facilitated in films with low thicknesses, which contributes to the action of soil microorganisms becoming faster and more effective, thus generating total biodegradation [51, 52].

**Table 3:** Determination of thickness of the films.***

Samples	Thickness (mm)
**CF** †	0.57 ± 0.14^a^
**AF1** ‡	0.63 ± 0.18^ab^
**AF2** §	0.71 ± 0.10^b^
**AF3** ¶	0.75 ± 0.26^b^

*** Each value represents the mean of 15 replicates with corresponding standard deviation. Data were statistically analyzed with multiple range test, using the method of least significant difference (LSD) Fisher. Different lowercase letters denote statistically significant differences (p < 0.05).

† CF: Control film (10% edible gelatin, 10% glycerol, 80% distilled water).

‡ AF1: Active film 1 (10% edible gelatin, 10% glycerol; 75% distilled water and 5% nisin/Na-EDTA solution).

§ AF2: Active film 2 (10% edible gelatin, 10% glycerol; 70% distilled water and 10% nisin/Na-EDTA solution).

¶ AF3: Active film 3 (10% edible gelatin, 10% glycerol; 60% distilled water and 20% nisin/Na-EDTA solution).

Thicker films are often not preferable because they appear bulky and take up more space than thinner films, resulting in higher transportation and storage cost [53].

**Fig 5:**
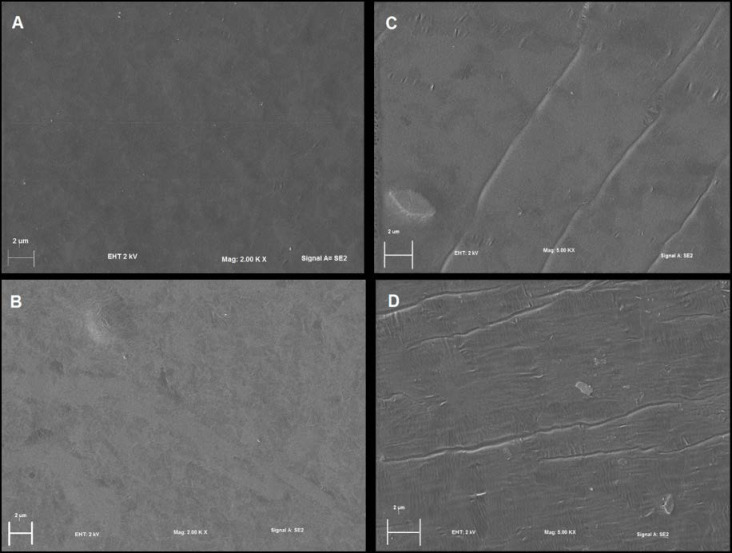
SEM micrograph of gelatin/nisin-EDTA films: A) CF; B) AF1; C) AF2 and D) AF3.

*Morphology of the films*. The surface morphology of gelatin and gelatin/nisin films are shown in Fig 5. CF sample shows a smooth and homogeneous surface, while the films with nisin incorporated show some roughness but without aggregation. The above indicates that the levels of nisin used did not alter the microstructure of the films [54]. It is observed that the morphology of the films containing nisin is different depending on the concentration of nisin used. AF3, which corresponds to the film with the highest contents of nisin, shows the roughest surface. Otherwise, AF1, which is the film with the lowest contents of nisin, shows a similar surface to the control sample. These micrographs showed that the nisin was incorporated into the gelatin matrix generating films of homogenous surface, which is indicative of good dispersion and compatibility between gelatin and nisin.

#### Biodegradation analysis of the films

The assays shown in Table 4 were kept under room temperature (20.0 ± 1.5°C) and RH (65.0 ± 4.0%), and the moisture of the composite was kept by spraying water once a day. The biodegradation analysis of the films was performed to simulate natural degradation conditions and to expose the films to the action of the mixed microbiota in the soil. The soil microbiota is rich concerning diversity of microorganisms, and when in contact with the humidity of the medium, it allows the degradation of the films. The incorporation of the antimicrobial agent into the gelatin 220 bloom films did not significantly affect their degradation capacity (p>0.05) and produced a 100% degradation, therefore, the results suggest that the active agent, in conjunction with gelatin, is susceptible to the rapid action of microorganisms and the biodegradation process. Similar results were reported by de Oliveira et al. [18], who performed biodegradability analysis of alginate films with cottonseed protein hydrolysates, obtaining 96 to 98% biodegradability in the same period of days of this assay (10 days). The biodegradation analysis of the films was performed to simulate natural degradation conditions and to expose the films to the action of the mixed microbiota in the soil. The soil microbiota is rich concerning diversity of microorganisms, and when in contact with the humidity of the medium, it allows the degradation of the films [55]. Films based on organic materials degrade easily and minimize environmental impact, hence the importance of biodegradation, which is a process characterized by weight loss. This process is favored if the thickness of the material is less than 10 mm since it allows bacterial activity to attack the amorphous areas (responsible for the elasticity), thus generating the total destruction of the macromolecular support of the polymer, and in parallel, of the by-products that are formed because of the reactions [16]. It should be noted that in the case of both gelatin-based films and alginate films, despite containing antimicrobial substances in their composition, the percentage of biodegradability is 100%. Moreover, research by Janani et al. [56] observed bioactive nanocomposite films composed of tragacanth (TG), polyvinyl alcohol (PVA), ZnO nanoparticles (NPs), and ascorbic acid (AA) using glycerol as a plasticizer and citric acid as a cross-linker for food packaging. Pure PVA and TG films showed the lowest and highest biodegradation rate of 5% and 100% after 60 days in this study. However, it was reported that PVA has a high resistance to biodegradation in the soil as its carbon backbone is not susceptible to biodegradation; hence, its slight weight loss is related to its characteristic hydrolyzing ability. At the same time, they point out that by decreasing the concentration of PVA and increasing the concentration of ZnO nanoparticles in the films, the percentages of biodegradability of the films are observed.

**Table 4:** Evaluation of the biodegradability percentage of the films.*

Sample	Biodegradability (% at 10 days)
**CF** †	100^a^
**AF1** ‡	100^a^
**AF2** §	100^a^
**AF3** ¶	100^a^

* Each value represents the mean of 3 replicates with the corresponding standard deviation. Data were statistically analysed with multiple range test, using the method of least significant difference (LSD) Fisher. Different lowercase letters denote statistically significant differences (p < 0.05).

† CF: Control film (10% edible gelatin, 10% glycerol, 80% distilled water).

‡ AF1: Active film 1 (10% edible gelatin, 10% glycerol; 75% distilled water and 5% nisin/Na-EDTA solution).

§ AF2: Active film 2 (10% edible gelatin, 10% glycerol; 70% distilled water and 10% nisin/Na-EDTA solution).

¶ AF3: Active film 3 (10% edible gelatin, 10% glycerol; 60% distilled water and 20% nisin/Na-EDTA solution).

The biodegradability of bio-based materials is the most important factor; it depends on the chemical structure, hydrophilicity, reactivity, and swelling behavior of polymeric chains. Other important factors are physical and physico-mechanical properties such as crystallinity, molecular weight, porosity, elasticity, and morphology [57].

#### Antimicrobial effect of nisin

The antimicrobial activity of the different films (CF, AF1, AF2, and AF3) was tested against *E*. *coli* ATCC 25922 at week 0 and 2 of elaboration. These time ranges were determined aiming to find a possible packaging system for fresh meat of direct sale, whose food-packaging contact period (film) would be around 5 days maximum. The antimicrobial action only occurs in the films with the antimicrobial agent and not in the control films. Regarding the freshly made films (t_0_), the growth of E. coli was reduced by approximately 3 logarithmic cycles in the active films (Table 5). This high antimicrobial activity is given by the content of nisin and Na-EDTA in the films, which was estimated above the minimum inhibitory concentration of this active mixture in order to inhibit *E*. *coli*. Then, a reduction in antimicrobial activity at week 2 (t_15_) is observed in the films, where the films with 5%, 10%, and 20% of the active agent, respectively, reduce approximately 1, 1, and 3 logarithmic cycles. In this regard, it should be noted that the outer membrane of the gram-negative bacteria acts as a permeability barrier for the cell and prevents nisin from reaching the cytoplasmic membrane due to the presence of phospholipids (interior) and lipopolysaccharides (exterior), which reduce the formation of hydrophobic compounds. [18, 58]. The lipopolysaccharides in the outer layer of the membrane have a negative charge and the central region of the oligosaccharide is ordered by cations (Ca^2+^ and Mg^2+^) that prevent nisin from reaching lipid II in the inner membranes [33]. To improve the antibacterial activity and action of nisin and the susceptibility of gram-negative bacteria to it, the penetration capacity of the membrane must first be improved to allow pore formation, thus allowing the mode of action of nisin [36, 59]. To destabilize the membrane and increase the activity of nisin, it is combined with Na-EDTA; the latter replaces the divalent cations of its binding sites (removes Mg^2+^ and Ca^2+^) and reduces the interaction between lipopolysaccharide molecules. The loss of lipopolysaccharides leads to the appearance of phospholipids on the outer membrane and the formation of channels through the pores, in which hydrophobic residues that allow the binding of nisin are found [60, 61].

**Table 5:** Evaluation of the antimicrobial capacity of the films.*

Time (week)	Logarithmic cycle reduction			
t_days_ = n°week	CF†	AF1‡	AF2§	AF3¶
**T**_**0**_ **= 0**	0^a^	3.15 ± 0.24^b^	3.26 ± 0.44^b^	3.80 ± 0.51^b^
**T**_**15**_ **= 2**	0^a^	1.19 ± 0.05^b^	1.11 ± 0.16^b^	2.95 ± 0.73^c^

* Each value represents the mean of 3 replicates with the corresponding standard deviation. Data were statistically analysed with multiple range test, using the method of least significant difference (LSD) Fisher. Different lowercase letters denote statistically significant differences (p < 0.05).

† CF: Control film (10% edible gelatin, 10% glycerol, 80% distilled water).

‡ AF1: Active film 1 (10% edible gelatin, 10% glycerol; 75% distilled water and 5% nisin/Na-EDTA solution).

§ AF2: Active film 2 (10% edible gelatin, 10% glycerol; 70% distilled water and 10% nisin/Na-EDTA solution).

¶ AF3: Active film 3 (10% edible gelatin, 10% glycerol; 60% distilled water and 20% nisin/Na-EDTA solution).

## Discussion

The results obtained agree with those from similar studies in which the effectiveness has been demonstrated by combining nisin and Na-EDTA to generate an antibacterial effect against Gram-negative bacteria. Ay and Tuncer [62] reported that combining nisin and EDTA to face *Salmonella typhimurium* SL 1344 approximately reduces 2 logarithmic cycles after 6 hours of incubation and between 0.7 to 0.96 logarithmic cycles after 24 hours of incubation, considering that this combination was in direct contact with the bacterial strain. Sangcharoen et al. [36], reported that the mixture of nisin + EDTA achieved a significant inhibitory effect on *Salmonella enteritis* ATCC 13076, reducing 1.45 logarithmic cycles when compared to the mixture of nisin + ascorbic acid + EDTA, which had a high inhibitory effect, reducing 3.41 logarithmic cycles; this is mainly explained by the reduction the pH facilitated by the addition of acid, which disables the growth of bacteria. Barís Bingol et al. [61] stated that the inhibitory activity of nisin occurs when it is combined with a chelating agent such as EDTA, generating inhibition against a wide variety of Salmonella species. With 30 minutes of exposure to 0.02M EDTA and 50 IU/mL of nisin, it is able to reduce 2.5 to 4.7 logarithmic cycles, and with 60 minutes of exposure, it reduces growth by 3.2 to 5.3 logarithmic cycles.

## Conclusion

Active films for inhibiting Escherichia coli were successfully obtained after the incorporation of nisin into the gelatin films, as a significant antimicrobial effect was found through in vitro assays. The incorporation of nisin was verified through FT-IR analysis and the TGA analysis showed the effects of the interactions concerning the thermal stability of gelatin films.

Gelatin films exposed in natural organic soil degraded rapidly (100%), which can be attributed to a higher microbial population (especially fungal biomass) and phosphorous availability. Soil acidity and phosphorous availability are generally the main factors responsible for the increase of fungi population. The film crystallinity could be affected by the soil moisture level, which is reduced in high moisture conditions. As a consequence, the hydrolysis rate of the polymer may be changed depending on the degree of disorder of the polymer. The biodegradability process of the films is favored in films with low thicknesses. At the same time, the thickness of the films also plays a fundamental role in the opacity values, as well as in their morphology, which was reflected in the SEM analysis.

The elaborated films would be viable for use in the packaging of fresh meat, since when in contact with *E*. *coli* at t_0_, they reduce 3 logarithmic cycles, which is relevant regarding direct sales products where the packaging has contact with food for periods of hours or a maximum of 4 to 5 days at refrigeration temperatures. In this way, the shelf life of this highly perishable type of food would be preserved. At the same time, the elimination of the packaging material becomes friendly to the environment, which is reaffirmed by the biodegradability analysis.

## References

[1] Vilela C, Kurek M, Hayouka Z, Rocker B, Yildirim S, Antunes M, et al. A concise guide to active agents for active food packaging: Review. Trends Food Sci Technol. 2018; 80: 212–222. doi: 10.1016/j.tifs.2018.08.006

[2] Marturano V, Bizarro V, Ambrogi V, Cutignano A, Tommonaro G, Abbamondi G, et al. Light-responsive nanocapsule-coated polymer films for antimicrobial active packaging. Polymers. 2019; 11(1): 68. doi: 10.3390/polym11010068 30960052 PMC6402017

[3] Dominguez R, Barba J, Gómez B, Putnik P, Bursac D, Pateiro M, et al. Active packaging films with natural antioxidants to be used in meat industry: A review. Food Res Int. 2018; 113: 93–101. doi: 10.1016/j.foodres.2018.06.073 30195551

[4] Schumann B, Schmid B. Packaging concepts for fresh and processed meat- Recent progresses. Innov Food Sci Emerg Technol. 2018; 47: 88–100. doi: 10.1016/j.ifset.2018.02.005

[5] Orhan-Yanikan E, Silva-Janeiro S, Ruiz-Rico M, Jimenez-Belenguer A, Ayhan K, Barat J. Essential oils compounds as antimicrobial and antibiofilm agents against strains present in the industry. Food Control. 2019; 101(2): 29–38. doi: 10.1016/j.foodcont.2019.02.035

[6] Osaili T, Hasan F, Kumar D, Obaid R, Al-Nabulsi A, Rao S, et al. Thermal inactivation of Escherichia coli O157:H7 strains and Salmonella spp. in camel meat burgers. LWT. 2020; 120: 108914. doi: 10.1016/j.lwt.2019.108914

[7] Yang X, Wang H, He A, Tran F. Biofilm formation and susceptibility to biocides of recurring and transient Escherichia coli isolated from meat fabrication equipment. Food Control. 2018; 90: 205–211. doi: 10.1016/j.foodcont.2018.02.050

[8] Li Y, Pei X, Zhang X, Wu L, Liu Y, Zhou H, et al. A surveillance of microbiological contamination on raw poultry meat at retail markets in China. Food Control. 2019; 104: 99–104. doi: 10.1016/j.foodcont.2019.04.037

[9] Projahn M, von Tippelskirch P, Semmler T, Guenther S, Alter T, Roesler U. Contamination of chicken meat with extended-spectrum beta-lactamase producing- Klebsiella pneumoniae and Escherichia coli during scalding and defeathering of broiler carcasses. Food Microbiol. 2019; 77: 185–191. doi: 10.1016/j.fm.2018.09.010 30297049

[10] Dairi N, Ferfera-Harrar H, Ramos M, Garrigós M. Cellulose acetate/ AgNPs- organoclay and/or thymol nano-biocomposite films with combined antimicrobial/antioxidant properties for active food packaging use. Int J Biol Macromol. 2019; 121: 508–533. doi: 10.1016/j.ijbiomac.2018.10.042 30321636

[11] Pascall M. The role and importance of packaging and labeling in assuring food safety, quality & compliance with regulations I: Packaging basics. In: Gordon A, editors. Food Safety and Quality Systems in Developing Countries. Academic Press; 2020. pp. 261–283.

[12] Williams H, Lindström A, Trischler J, Wikström F, Rowe Z. Avoiding food becoming waste in households–The role of packaging in consumers’ practices across different food categories. J Clean Produ. 2020; 256: 121775. doi: 10.1016/j.jclepro.2020.121775

[13] Kaur S, Puri D. Active and intelligent packaging: A boon to food packaging. Int J Food Sci Nutr. 2017; 2: 15–18.

[14] Cazón P, Vásquez M, Velásquez G. Cellulose-glycerol-polyvinyl alcohol composite films for food packaging: evaluation of water adsorption, mechanical properties, light-barrier properties and transparency. Carbohydr Polym. 2018; 195: 432–443. doi: 10.1016/j.carbpol.2018.04.120 29804996

[15] Khan B, Khan M, Samin G, Jahan Z. Thermoplastic starch: A possible biodegradable food packaging material- A review. J Food Process Eng. 2016; 40(3): e12447 doi: 10.1111/jfpe.12447

[16] Suderman N, Isa M, Sarbon N. The effect of plasticizers on the functional properties of biodegradable gelatin- based film: A review. Food Biosci. 2018; 24(3): 111–119. doi: 10.1016/j.fbio.2018.06.006

[17] Ketelsen M, Janssen M, Hamm U. Consumers’ response to environmentally friendly food packaging—A systematic review. J Clean Prod. 2020: 254(4); 120123. doi: 10.1016/j.jclepro.2020.120123

[18] de Oliveira J, Moraes J, Fernandes A, Borges de Almeida A, Mayara de Lima T, Pereira K, et al. Active food packaging: Alginate films with cottonseed protein hydrolysates. Food Hydrocoll. 2019; 92: 267–275. doi: 10.1016/j.foodhyd.2019.01.052

[19] Ahammed S, Liu F, Khin M, Yokoyama W, Zhong F. Improvement of the water resistance and ductility of gelatin film by zein. Food Hydrocoll. 2020; 105(1): 105804. doi: 10.1016/j.foodhyd.2020.105804

[20] Basha M. Optical properties and colorimetry of gelatin gels prepared in different saline solutions. J Adv Res. 2019;16: 55–65. doi: 10.1016/j.jare.2018.12.002 30899589 PMC6412942

[21] Popović S, Vera L, Hromiš N, Šuput D, Bulut D. Biopolymer Packaging Materials for Food Shelf-Life Prolongation. In: Grumezescu AM, Holban AM, editors. Handbook of Food Bioengineering, Biopolymers for Food Design. Academic Press; 2018. pp. 223–277.

[22] Garavand H, Rouhi M, Razavi S, Cacciotti I. Mohammadi R. Improving the integrity of natural biopolymer films used in food packaging by crosslinking approach: A review. Int J Biol Macromol. 2017; 104: 687–707. doi: 10.1016/j.ijbiomac.2017.06.093 28652152

[23] Zhong Y, Godwin P, Jin Y, Xiao H. Biodegradable polymers and green-based antimicrobial packaging materials: A mini-review. Adv Ind Eng Polym Res. 2020; 3(1): 27–35. doi: 10.1016/j.aiepr.2019.11.002

[24] Singh S, Ho Lee M, Park L, Shin Y, Lee Y. Antimicrobial seafood packaging: A review. J Food Sci Technol. 2016; 53(6): 2505–2518. doi: 10.1007/s13197-016-2216-x 27478206 PMC4951407

[25] Tyuftin A, Kerry J. Review of surface treatment methods for polyamide films for potential application as smart packaging materials: surface structure, antimicrobial and spectral properties. Food Packag Shelf Life. 2020; 24: 100475. doi: 10.1016/j.fpsl.2020.100475

[26] El Fawal G, Hong H, Song X, Wu J, Sun M, He C, et al. Fabrication of antimicrobial films based on hydroxyethylcellulose and ZnO for food packaging application. Food Packag Shelf Life. 2020; 23: 100462. doi: 10.1016/j.fpsl.2020.100462

[27] Santos J, Sousa R, Otoni C, Moraes A, Souza V, Medeiros E, et al. Nisin and other antimicrobial peptides: Production, mechanisms of action, and application in active food packaging. Innov Food Sci Emerg Technol. 2018; 48: 179–194. doi: 10.1016/j.ifset.2018.06.008

[28] Campion A, Morrissey R, Field D, Cotter P, Hill C, Ross P. Use of enhanced nisin derivatives in combination with food-grade oils or citric acid to control Cronobacter sakazakii and Escherichia coli O157:H7. Food Microbiol. 2017; 65: 254–263. doi: 10.1016/j.fm.2017.01.020 28400011

[29] Ajingi Y, Ruengvisesh S, Khunrae P, Rattanarojpong T, Jongruja N. The combined effect of formic acid and Nisin on potato spoilage. Biocatal Agric Biotechnol. 2020; 24: 101523. doi: 10.1016/j.bcab.2020.101523

[30] Prudêncio CV, Mantovani HC, Cecon PR, Prieto M, Vanetti MCD. Temperature and pH influence the susceptibility of Salmonella Typhimurium to nisin combined with EDTA. Food Control. 2016; 61: 248–253. 10.1016/j.foodcont.2015.09.042.

[31] Moradi M, Kousheh SA, Almasi H, Alizadeh A, Guimarães JT, Yılmaz N, et al. Postbiotics produced by lactic acid bacteria: The next frontier in food safety. Compr Rev Food Sci Food Saf. 2020; 19(6): 3390–3415. doi: 10.1111/1541-4337.12613 33337065

[32] Abarca R, Rodríguez F, Guarda A, Galotto M, Bruna J, Fávaro Pérez M, et al. Application of β-Cyclodextrin/2-Nonanone Inclusion Complex as Active Agent to Design of Antimicrobial Packaging Films for Control of Botrytis cinerea. Food Bioproc Tech. 2017; 10(9): 1585–1594. doi: 10.1007/s11947-017-1926-z

[33] Zhou L, Van Heel A, Montalban-Lopez M, Kuipers P. Potentiating the activity of nisin against *Escherichia coli*. Front Cell Dev Biol. 2016; 4(7): 9. doi: 10.3389/fcell.2016.00007 26904542 PMC4745983

[34] Clinical and Laboratory Standards Institute (CLSI). Performance Standards for Antimicrobial Susceptibility Testing CLSI Supplement M100S. 26th ed. Wayne: Clinical and Laboratory Standards Institute; 2018.

[35] Van Den Honert M, Gouws P, Hoffman L. A Preliminary Study: Antibiotic Resistance Patterns of Escherichia coli and Enterococcus Species from Wildlife Species Subjected to Supplementary Feeding on Various South African Farms. Animals. 2020; 10(3): 396. doi: 10.3390/ani10030396 32121124 PMC7142571

[36] Sangcharoen N, Klaypradit W, Wilaipun P. Antimicrobial activity optimization of nisin, ascorbic acid and ethylenediamine tetraacetic acid disodium salt (EDTA) against Salmonella Enteritidis ATCC 13076 using response surface methodology. Agric Nat Resour. 2017; 51(5): 355–364. doi: 10.1016/j.anres.2017.12.005

[37] Roy S, Rhim J-W. Preparation of antimicrobial and antioxidant gelatin/curcumin composite films for active food packaging application. Colloids Surf B: Biointerfaces. 2020; 188: 110761–110768. doi: 10.1016/j.colsurfb.2019.110761 31901685

[38] Chentir I, Kchaou H, Hamdi M, Jridi M, Li S, Doumandji A, et al. Biofunctional gelatin-based films incorporated with food grade phycocyanin extracted from the Saharian cyanobacterium Arthrospira sp. Food Hydrocoll. 2019; 89: 715–725. doi: 10.1016/j.foodhyd.2018.11.034

[39] Hubner P, Donati N, de Menezes Quines LK, Tessaro IC, Marcilio NR. Gelatin-based films containing clinoptilolite-Ag for application as wound dressing. Mater Sci Eng C Mater Biol Appl. 2020; 107: 110215–110229. doi: 10.1016/j.msec.2019.110215 31761173

[40] Kumar S, Shukla A, Baul P, Mitra A, Halder D. Biodegradable hybrid nanocomposites of chitosan/gelatin and silver nanoparticles for active food packaging applications. Food Packag Shelf Life. 2018; 16: 178–184. doi: 10.1016/j.fpsl.2018.03.008

[41] Sabo B, Becica T, Keles N, Kovacevic D, Brozovic M. The impact of packaging transparency on product attractiveness. Journal of Graphic Engineering and Desing. 2017; 8(2): 5–9. doi: 10.24867/jged-2017-2-005

[42] Andreuccetti C, Carvalho R, Grosso C. Effect of hydrophobic plasticizers on functional properties of gelatin-based films. Food Res Int. 2009; 42(8): 1113–1121. doi: 10.1016/j.foodres.2009.05.010

[43] Mishra R, Varshney R, Das N, Sircar D, Roy P. Synthesis and characterization of gelatin-PVP polymer composite scaffold for potential application in bone tissue engineering. Eur. Polym. J. 2019; 119(6): 155–168. doi: 10.1016/j.eurpolymj.2019.07.007

[44] Kong J, Yu S. Fourier transform infrared spectroscopic analysis of protein secondary structures. Acta Biochim Biophys Sin. 2007; 39: 549–559. doi: 10.1111/j.1745-7270.2007.00320.x 17687489

[45] Jackson M, Choo LP, Watson PH, Halliday WC, Mantsch HH. Beware of connective tissue proteins: Assignment and implications of collagen absorptions in infrared spectra of human tissues. Biochim BiophysActa. 1995; 1270: 1–6. doi: 10.1016/0925-4439(94)00056-v 7827129

[46] Gong F, Qian J, Chen Y, Yao S, Tong J, Guo H. Preparation and properties of gum arabic cross-link binding nisin microparticles. Carbohydr Polym. 2018; 197: 608–613. doi: 10.1016/j.carbpol.2018.05.080 30007653

[47] Wu T, Wu C, Fang Z, Ma X, Chen S, Hu Y. Effect of chitosan microcapsules loaded with nisin on the preservation of small yellow croaker. Food Control. 2017; 79: 317–324. doi: 10.1016/j.foodcont.2017.04.016

[48] Chopra M, Kaur P, Bernela M, Thakur R. Surfactant assisted nisin loaded chitosan-carageenan nanocapsule synthesis for controlling food pathogens. Food Control. 2014; 37: 158–164. doi: 10.1016/j.foodcont.2013.09.024

[49] Riaz A, Lei S, Saleem H, Wan P, Chen D, Jabbar S, et al. Preparation and characterization of chitosan-based antimicrobial active food packaging film incorporated with apple peel polyphenols. Int J Biol Macromol. 2018; 114: 547–555. doi: 10.1016/j.ijbiomac.2018.03.126 29578019

[50] Zuo G, Song X, Chen F, Shen Z. Physical and structural characterization of edible bilayer films made with zein and corn-wheat starch. J Saudi Soc Agric Sci. 2019; 18(3): 324–331. doi: 10.1016/j.jssas.2017.09.005

[51] Thakur S, Chaudhary J, Sharma B, Verma A, Tamulevicius S, Thakur V. Sustainability of bioplastics: Opportunities and challenges. Curr Opin Green Sustain Chem. 2018; 13: 68–75. doi: 10.1016/j.cogsc.2018.04.013

[52] Rech C, da Silva K, Bagnara B, Stival P, Turim M, Serantoni T, et al. Biodegradation of polyhydroxybutyrate films incorporated with eugenol in different soil types. CSCEE. 2020; 2: 100014. doi: 10.1016/j.cscee.2020.100014

[53] Syahida S, Ismail-Fitry M, Asa’ari Ainun Z, Nur Hanani Z. Effects of palm wax on the physical, mechanical and water barrier properties of fish gelatin films for food packaging application. Food Packag Shelf Life. 2020; 23: 100437. doi: 10.1016/j.fpsl.2019.100437

[54] Zimet P, Mombrú AW, Mombrú D, Castro A, Villanueva JP, Pardo H, et al. Physico-chemical and antilisterial properties of nisin-incorporated chitosan/carboxymethyl chitosan films. Carbohydr Polym. 2019; 219; 334–343. doi: 10.1016/j.carbpol.2019.05.013 31151533

[55] da Silva G, Romani V, Guimaraes V. Biodegradable and active-intelligent films based on methylcellulose and jambolão (Syzygium cumini) skins extract for food packaging. Food Hydrocoll. 2020; 109: 106139. doi: 10.1016/j.foodhyd.2020.106139

[56] Janani N, Zare EN, Salimi F, Makvandi P. Antibacterial tragacanth gum-based nanocomposite films carrying ascorbic acid antioxidant for bioactive food packaging. Carbohydr Polym. 2020; 247: 116678. doi: 10.1016/j.carbpol.2020.116678 32829806

[57] Jha P. Effect of plasticizer and antimicrobial agents on functional properties of bionanocomposite films based on corn starch-chitosan for food packaging applications. Int J Biol Macromol. 2020; 160: 571–582. doi: 10.1016/j.ijbiomac.2020.05.242 32485256

[58] Khan A, Dang Vu K, Riedl B, Lacroix M. Optimization of the antimicrobial activity of nisin, Na-EDTA and pH against gram-negative and gram-positive bacteria. Food Sci Technol. 2015; 61(1): 124–129. 10.1016/j.lwt.2014.11.035.

[59] Morsy M, Elsabagh R, Trinetta V. Evaluation of novel synergistic antimicrobial activity of nisin, lysozyme, EDTA nanoparticles, and/or ZnO nanoparticles to control foodborne pathogens on minced beef. Food Control. 2018; 92: 249–254. doi: 10.1016/j.foodcont.2018.04.061

[60] Field D, Baghou I, Rea M, Gardiner G, Ross R, Hill C. Nisin in combination with cinnamaldehyde and EDTA to control growth of Escherichia coli strains of swine origin. Antibiotcs. 2017; 6(4): 35. doi: 10.3390/antibiotics6040035 29231854 PMC5745478

[61] Baris Bingol E, Akkaya E, Hampikyan H, Cetin O, Colak H. Effect of nisin-EDTA combinations and modified atmosphere packaging on the survival of Salmonella enteritidis in Turkish type meatballs. CyTA J Food. 2018; 16: 1030–1036. doi: 10.1080/19476337.2018.1523810

[62] Ay Z, Tuncer Y. Combined Antimicrobial Effect of Nisin, Carvacrol and EDTA against Salmonella typhimurium in TSBYE at 4°C and 37°C. Rom Biotech Lett. 2016; 21(4): 11666–11674.

